# Investigation on forced vibration characteristics of Nitinol tracheal stent

**DOI:** 10.1186/s12938-022-01054-y

**Published:** 2022-12-10

**Authors:** Yu dong Bao, Sheng qian Qu, Wen Wei, Xun Li

**Affiliations:** 1 Harbin University of Science and Technology, Harbin, China; 2 Key Laboratory of Advanced Manufacturing and Intelligent Technology, Ministry of Education, Harbin, China; 3 K The Sixth Affiliated Hospital of Harbin Medical University, Harbin, China

**Keywords:** Tracheal stent, Periodic vibration, Random vibration, Stent loss

## Abstract

**Background:**

Tracheal stents can be placed in a narrow position in the human trachea to ensure smooth breathing. And the stent will deform during service by the influence of the physiological environment or random excitations, such as coughing.

**Methods:**

This paper divides the vibration into periodic and random vibrations according to the different pressures. And a coupling vibration model was established by analyzing the contact relationship between the stent and the trachea tissue. And this study discusses the influence of tracheal diameter, respiratory pressure, and frequency on the stent vibration characteristics through Ansys simulation. In addition, the nonlinear equations were solved by the Matlab numerical analysis method, which could help analyze the influence of cough intensity on the stability of the tracheal stent system.

**Results:**

The results showed that when tracheal stenosis occurred in the trachea's more significant grade, the trachea stent was more likely to fall off when treated with a tracheal stent. With the increase in respiratory frequency and pressure, the deformation of the tracheal stent is more considerable. Moreover, the frequency of normal cough hardly affects the stability of the stent system, while the excitation force and damping coefficient value greatly influence the system. When the excitation force of the cough exceeds the critical importance of 20 N, the tracheal stent is prone to fall off. This study comprehensively obtained the forced vibration characteristics of the stent under service conditions, which could make up for the shortage of the vibration theory of the stent.

**Conclusion:**

The results can provide a theoretical basis for predicting the possibility of stent loss in clinical treatment.

## Introduction

In recent years, with developing urbanization and industrialization, the atmosphere has been seriously polluted, leading to a rapid increase in the morbidity and mortality of respiratory diseases [1]. As a common respiratory disease, tracheal stenosis can lead to dyspnea and even asphyxia. Tracheal resection and reconstruction are often used in the traditional treatment for tracheal lesions, but surgery will bring great pain to patients and lead to human rejection [2–4]. With the popularization and development of bronchoscopy, doctors can determine the location of the lesion and judge the degree of stenosis under endoscopy. The stent is then pushed to the lesion site and released to provide lasting support [5–7]. Tracheal stenting can rapidly relieve dyspnea and improve pulmonary function in patients with airway stenosis [8, 9]. When the tracheal stent is implanted in the lesion location, it will be affected by the complex physiological environment in the body. For example, transpulmonary pressure acting on the airway wall during breathing causes radial deformation of the stent [10]. In addition, random stimulation, such as coughing and sneezing caused by external stimuli, can easily lead to stent shedding and then damage the inner wall of the trachea tissue. Therefore, studying the forced vibration characteristics of the stent under different conditions and pressures during service can help doctors better judge the stability of the stent. This study can provide a theoretical basis for optimizing stents in clinical practice and preventing stent loss.

Nitinol tracheal stent is widely used in clinical treatment as a medical stent with excellent performance [11]. The key to analyzing the vibration characteristics of the stent is to understand the properties of Nitinol and the mechanism of interaction between the stent and the tracheal wall. In recent years, many scholars have done some research on the vibration performance of the stent. In the field of studying the influence of tracheal deformation on stent performance, Malve et al. [12] established a finite element model of the human trachea, analyzed the deformation characteristics of the trachea wall during coughing, and modeled the trachea wall as a kind of superelastic solid material to simulate the implantation process of the endotracheal prosthesis. McGrath et al. [13] created a biomechanical lung model to evaluate the interaction between the stent and the airway, including various loading conditions, such as normal breathing, cough, and ventilation. The results showed that the airway deformation caused by tracheal movement significantly affects the stent's mechanical properties. Freitag et al. [14] conducted dynamic and static load simulation on isolated human trachea with a stent to study the stent's stability in trachea deformation. In the field of studying the nonlinearity and shedding mechanism of Ni–Ti alloy stents, Guo et al. [15] established the nonlinear dynamic response of shape memory alloy tracheal stents by considering the influence of random excitation, such as random radial pressure. And they discussed the impact of stent parameters on dynamic stent characteristics and determined the system parameter value when the stent fell off. Tao et al. [16] established the nonlinear mechanical model of Ni–Ti shape memory alloy tracheal stent structure and deduced the displacement and deformation equation. And they verified the geometric nonlinear mechanical model by numerical methods. Wang et al. [17] proposed a mechanical model of SMA tracheal stent in the process of cough and analyzed its dynamic response. The results showed that the vibration with a large amplitude could be eliminated by selecting the material with appropriate parameters. In addition, Ma et al. [18] proposed a mathematical model for the respiratory vibration problem of vascular stents. They used the Flugge shell theory to calculate the natural frequency of stent vibration.

In summary, many scholars’ studies on the vibration characteristics of Nitinol tracheal stents were primarily based on theoretical model analysis. The finite element method was not used to describe the vibration displacement response of the stent under various load conditions, such as normal breathing and coughing. Most existing studies on stent vibration focused on the deformation of the trachea wall or the dynamic response of the stent during cough. Still, they did not consider the trachea and stent as a system, and there was also a lack of vibration characteristics analysis of the stent under other breathing conditions. Because the pressure produced when people breathe periodic changes, the amplitude of cough will fluctuate. Therefore, the vibration of the tracheal stent is divided into periodic vibration and random vibration for analysis in this study. This paper has analyzed the radial deformation of the stent under different conditions, and the safety performance under random vibration, such as coughing, is also considered. This study comprehensively obtained the stent's forced vibration characteristics under service conditions to make up for the shortage of the stent vibration theory. The results can guide the selection and improvement of clinical stents and provide a theoretical basis for the prevention of stent shedding. It has great significance for the development of the stent industry.

## Results

In this paper, the commercial software Ansys is used to solve the periodic vibration of the tracheal stent coupling system. And the radial displacement of the stent was observed under different breathing conditions. Secondly, Matlab numerical simulation method was used to analyze the random vibration dynamic characteristics of the stent. And the influence of vibration frequency, amplitude, and damping coefficient of excitation force on vibration were considered. Moreover, the modal analysis of different diameter stents was carried out to judge the resonance situation under random excitation, such as cough. This study comprehensively obtained the vibration characteristics of stents under different impact loads. It could provide a theoretical basis for preventing scaffold shedding in clinical practice.

### Periodic vibration simulation analysis

The completion of human respiratory movement mainly depends on the effect of respiratory muscles on the chest. Since most of the bronchi are in the lung, transpulmonary pressure will directly act on the outer wall of the trachea, resulting in the trachea contraction during breathing. When the stent is inserted into the diseased location, it will also move along with the trachea. Once the radial displacement of the stent is too large, it will fall off, seriously affecting the patient's safety. The human is in normal breathing most of the time. The breathing rate is about 10–20 times per minute, and the airway pressure is about 0.002 MPa. When inflammation occurs in the trachea or is stimulated by external factors, it will make the airway spasm so that the patient will appear shortness of breath symptoms. The respiratory rate is 20–30 times/min, and the airway pressure is about 0.004 MPa. When the human body carries out strenuous exercise or breathing, the respiratory rate is 30–40 times /min, and the airway pressure is about 0.007 MPa [19]. Because there are many tracheal grades in the human body, the treatment of different grades of trachea requires different diameter stents. This will affect the vibration characteristics of stents during breathing.

**Fig. 1:**
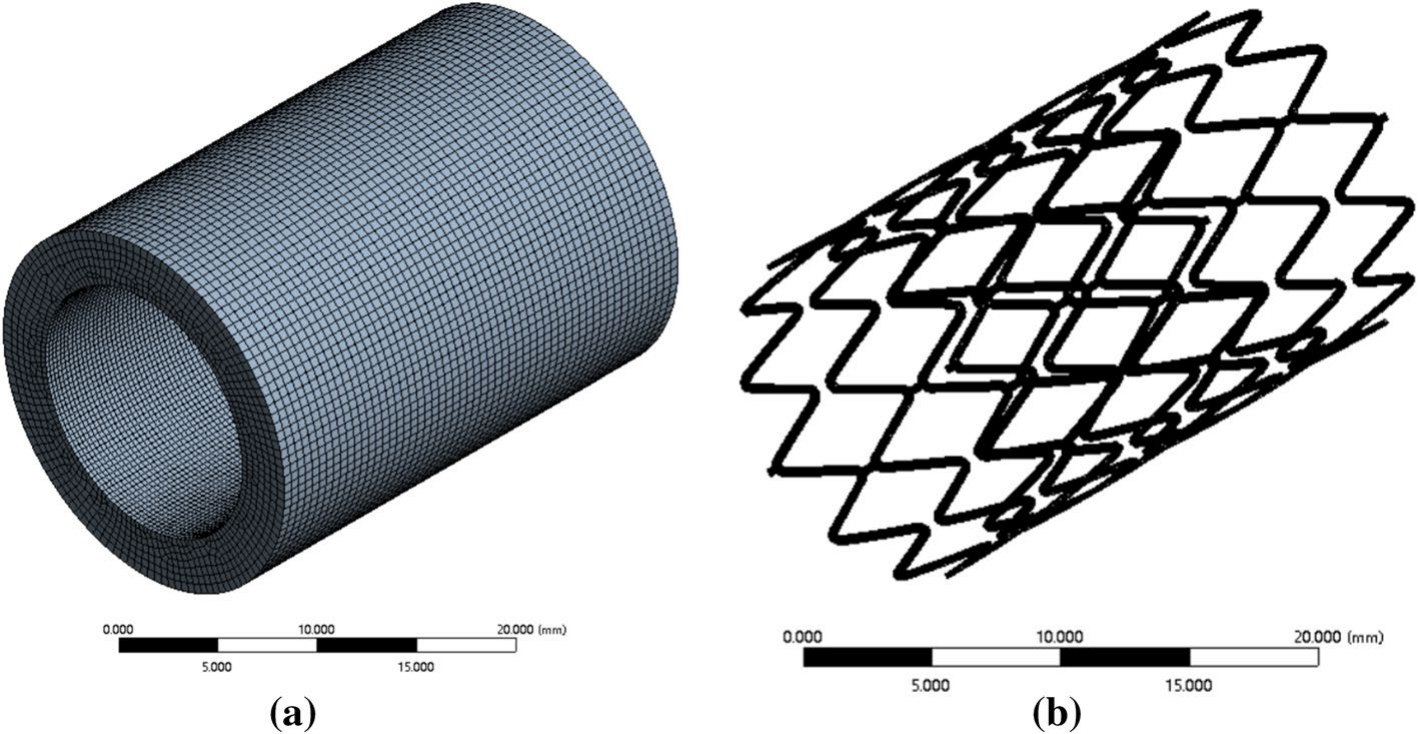
Grid model of the trachea and stent **a**. Tracheal tissue **b** Tracheal stent

According to Table 2, models of the stent and trachea were established in Solidworks software, the diameters of the three stents were 12, 13, and 14 mm, respectively, the length was 24 mm, and the thickness was 0.2 mm. The trachea model was a cylinder model with a wall thickness of 3 mm, a length of 28 mm, and an outer diameter of 12–14 mm. The model was saved as Step file format and imported into Ansys, and the transient dynamics module was used for simulation analysis. To simplify the analysis, the material density of trachea tissue was set as 0.5 g/cm^3^ and the elastic modulus is 5.1 MPa [20]. Besides, the Mooney-Rivlin model was used for parameter fitting, the C_01_ was set as 0.37085, C_10_ as − 0.3445, and Poisson's ratio as 0.47. As Ansys software has built-in memory alloy material, the material Settings of the stent are shown in Table 1 [21]. The tracheal tissue was meshed by hexahedral and adaptive mesh, and the mesh size was 0.5. To ensure the accuracy of the calculation, mesh refinement was performed on the area where the inner wall of the trachea contacted the stent, as shown in Fig. 1a. In addition, the unstructured tetrahedral mesh was used to partition the stent, and the mesh size was 0.5; the results are shown in Fig. 1b. The outer surface of the stent was set to be in contact with the inner wall of the trachea, and a periodically changing radial force was applied to the outer wall of the trachea to simulate the pressure generated during respiration. The boundary conditions were set as the rotation of the fixed stent, and the radial degrees of freedom were preserved.

**Table 1:** The parameters of the Ni–Ti material subroutine

Nitinol material properties	Data
Nitinol material density *ρ* (kg/m^3^)	6450
Elastic modulus of austenite phase *E*_A_ (MPa)	67000
Austenitic phase Poisson’s ratio *V*_A_	0.33
Elastic modulus of martensite phase *E*_M_(MPa)	26300
Martensitic phase Poisson’s ratio *V*_M_	0.33
Transformation strain $\varepsilon$_*L*_	0.067
(*δ*σ/*δ*T)_*L*_ (MPa T^−1^)	63.8
Initial stress of loading${\upsigma }_{\text{L}}^{\text{S}}$ (MPa)	200
End stress of loading ${\upsigma }_{\text{L}}^{\text{E}}$ (MPa)	300
Reference temperature T_0_(°C)	73
(*δσ/δ*T)_*U*_ (MPa T^−1^)	8
Initial stress of unloading ${\upsigma }_{\text{U}}^{\text{S}}$ (MPa)	100
End stress of unloading ${\upsigma }_{U}^{E}$ (MPa)	50
${\upsigma }_{\text{CL}}^{\text{S}}$(MPa)	200

### Effect of respiratory rate on vibration characteristics

**Fig. 2:**
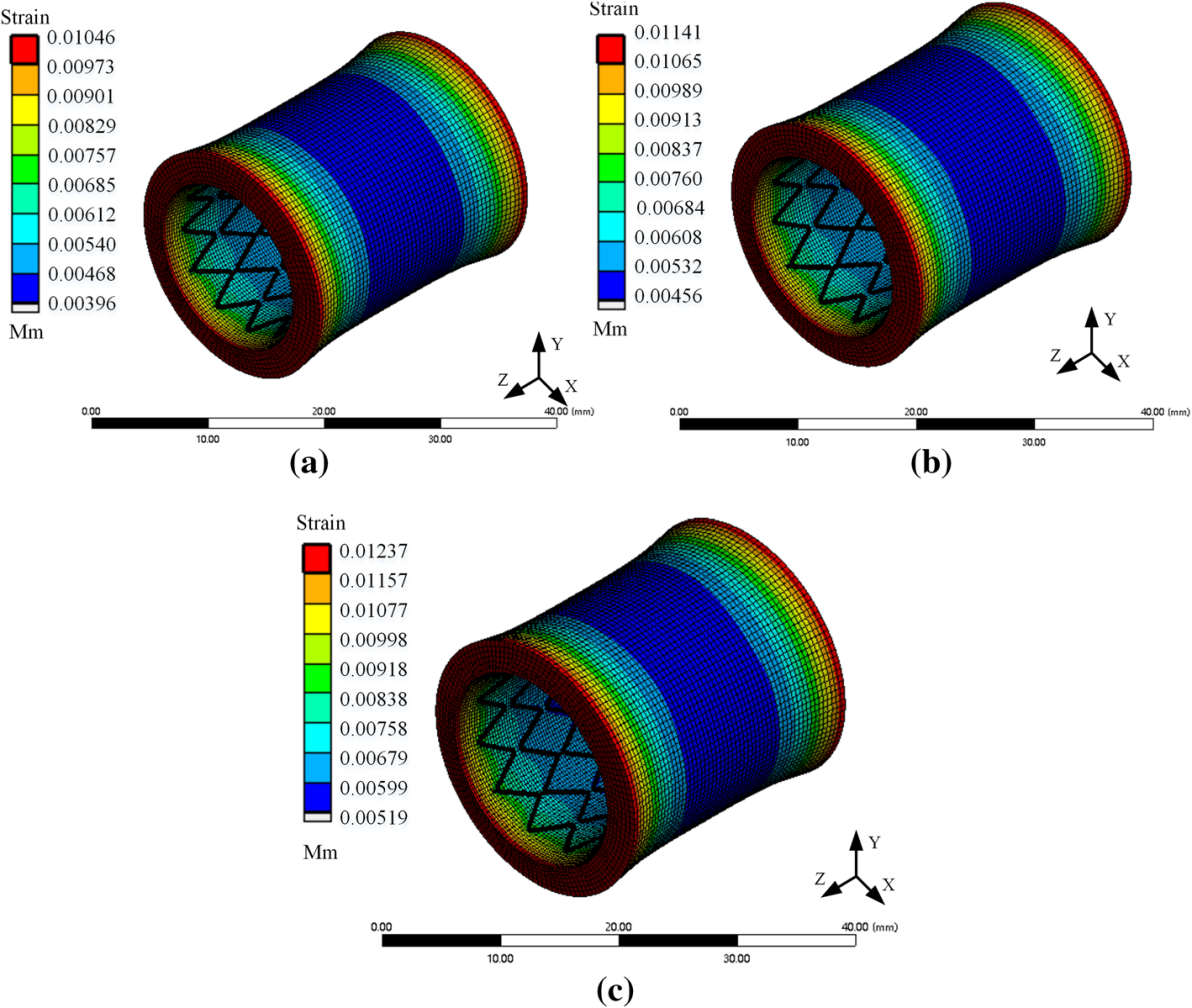
Total deformation program of tracheal—stent coupling model **a**. Diameter of 12 mm **b** Diameter of 13 mm **c** Diameter of 14 mm

**Fig. 3:**
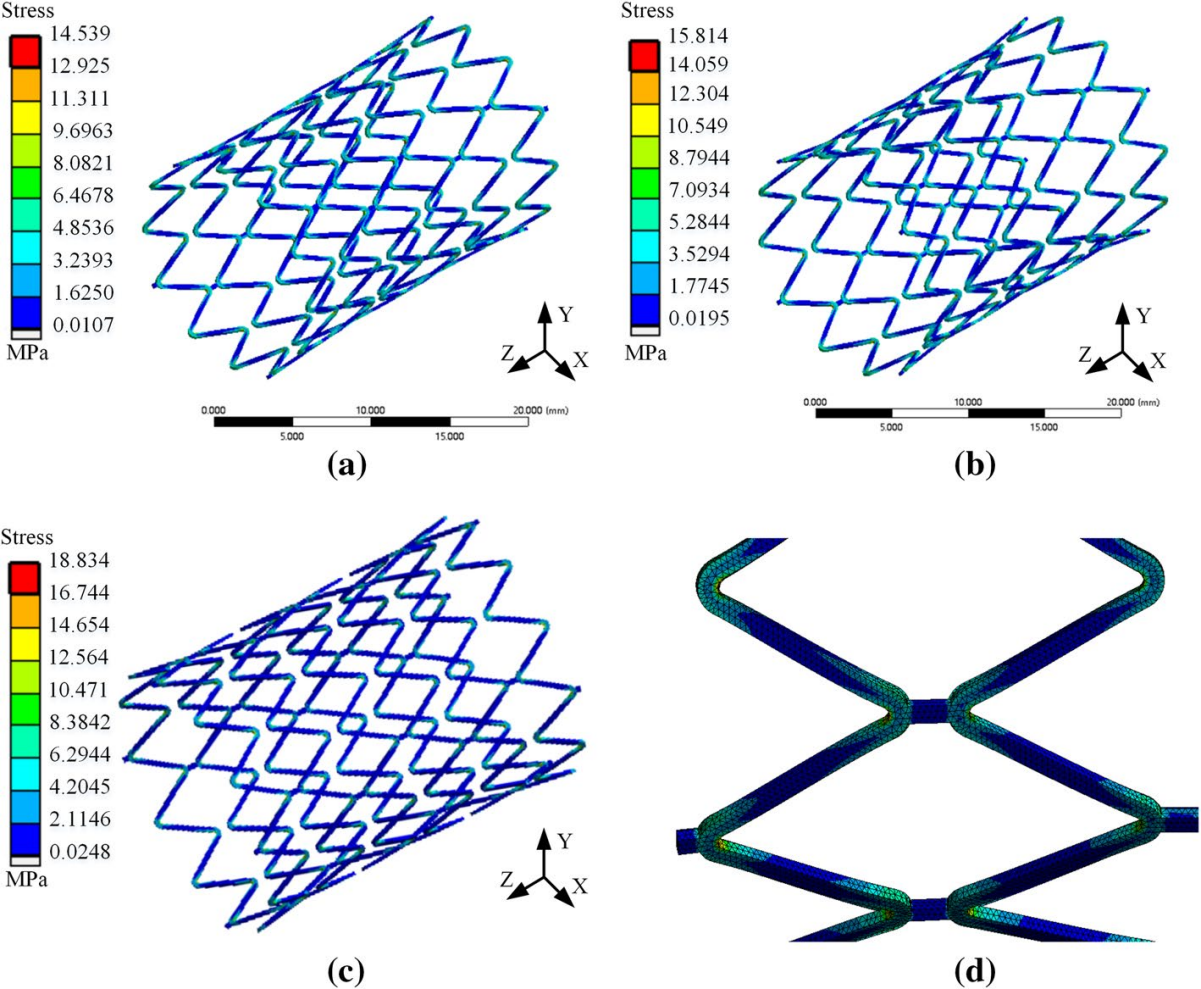
Stress cloud diagram of the stent **a**. The diameter of the trachea is 12 mm **b**. The diameter of the trachea is 13 mm **c**. The diameter of the trachea is 14 mm **d** Stress diagram of the local stent

The trachea is an essential part of the respiratory system, which is divided into the main bronchus and the right main bronchus. Each level of the trachea shows a bifurcation structure, extending to the alveoli [22]. Due to a large number of big tracheal furcation, the diameter of different levels of the trachea is not the same, so the treatment of tracheal stenosis should use different stents according to the actual condition. The influence of trachea diameter on stent loss was simulated by finite element analysis under normal breathing conditions. The respiratory rate was set as 0.3 Hz, the wall pressure was about 0.002mpa, and the diameter of the trachea was 12, 13, and 14 mm, respectively. A periodic pressure with a peak value of 0.002 MPa was applied to the tracheal wall, and the total deformation of the coupling system was obtained, as shown in Fig. 2. The stress distribution cloud diagram of the stent is shown in Fig. 3. It could be seen from Fig. 2 that when the human body was breathing normally, with the increase of the trachea diameter, the deformation of the trachea gradually increased. And the maximum displacement showed a decreasing trend from outside to inside. In addition, it can be seen from Fig. 3 that the larger the diameter of the tracheal stent, the greater the internal stress generated during its deformation. Figure 3d shows that the pressure on the stent was often concentrated at the bending of the pillar unit, while the pressure on the middle part and the connection was small. This indicated that tissue hyperplasia was easy to occur at both ends of the stent.

**Fig. 4:**
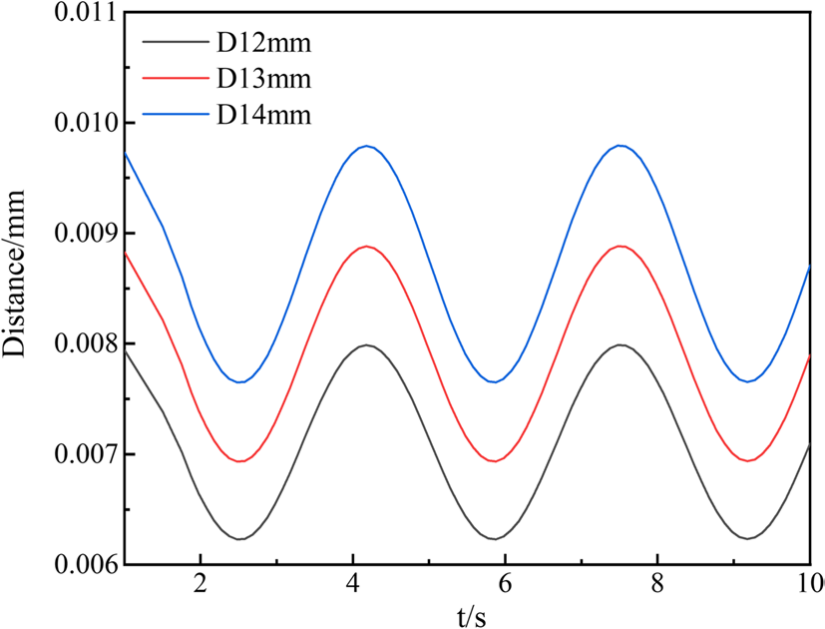
Radial displacement history of tracheal stents with different diameters

Based on the cylindrical coordinate system, the radial displacement data of the tracheal stent were extracted and fitted to obtain the radial deformation curve of the stent over time, as shown in Fig. 4.

It could be seen from Fig. 4 that the pressure acting on the tracheal wall changed periodically during normal breathing. Therefore, the radial displacement of the stent also showed a rare change form of fluctuation up and down. And the fluctuation frequency of different diameter tracheal stents was the same. As the trachea's diameter increased, the stent's radial displacement also gradually increased. The maximum radial displacement of the different diameter stents was 0.0079 mm, 0.0089 mm, and 0.0098 mm. This is because the radial support performance of the larger diameter bracket to the gas pipe wall was barely satisfactory. Moreover, the contact stress between the support and the tracheal wall was smaller. Therefore, when the tracheal wall was under tremendous pressure, it was relatively easy to deformation, resulting in stent loss. In conclusion, when the diameter of the diseased trachea is larger, stent loss is more likely to occur during the treatment. Therefore, the support performance of larger stents should be optimized in clinical medicine.

### Effect of respiratory pressure on vibration characteristics

**Fig. 5:**
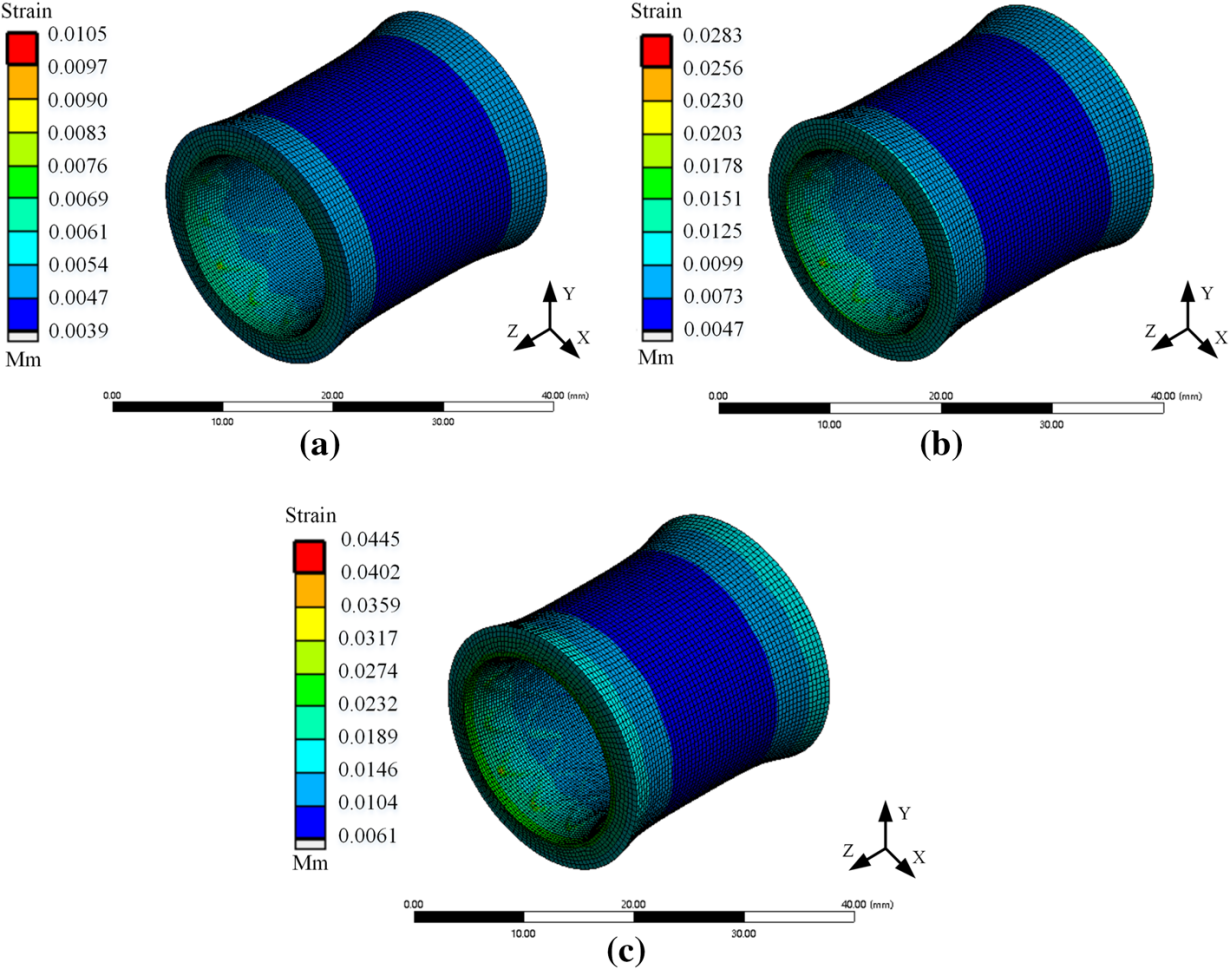
Vibration characteristics of the stent under different respiratory pressure **a** Pressure of 0.002 MPa **b** Pressure of 0.004 MPa **c** Pressure of 0.007 MPa

**Fig. 6:**
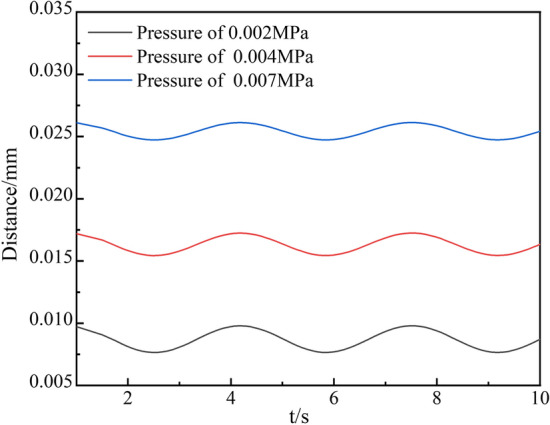
Displacement curves of the stent under different respiratory pressures

Patients often have different breathing conditions during the rehabilitation of stent implantation, such as rapid breathing and intense breathing caused by emotional agitation or allergic reactions. High-intensity breathing frequently produces large pressure on the trachea, affecting the stent's stability during service. To study the influence of pressure generated by different breathing states on the vibration characteristics of the stent, a 14 mm diameter stent was selected for vibration analysis at the normal respiratory rate (0.3 Hz) in this section. Periodic pressures with amplitudes of 0.002 MPa, 0.004 MPa, and 0.007 MPa were applied to the tracheal wall, so the equivalent stress program of the contact between the trachea and the stent was obtained, as shown in Fig. 5. The radial displacement of the stent was extracted for curve drawing, and the result is shown in Fig. 6.

Figure 5 shows that with the increase in respiratory pressure, the contact force between the tracheal wall and the support gradually increased. In addition, the stress was concentrated in the contact area between the front and ends of the stent and the tracheal wall. This indicated that the damage caused by stent movement to tracheal tissue was prone to occur at the end of the contact area, which was also the common area for tissue hyperplasia. Figure 6 shows that with the increase in respiratory pressure, the amplitude of tracheal stent fluctuation decreases, and the radial displacement of tracheal stent increases. The maximum values were 0.0098 mm, 0.0172 mm, and 0.0261 mm, respectively. It was obvious that when a patient had rapid breathing, such as asthma, or strenuous exercise, there was tremendous pulmonary pressure in the lungs. Moreover, it would compress the tracheal wall to produce large deformation so that the tracheal bracket was possible to lose.

### Effect of respiratory pressure on vibration characteristic

**Fig. 7:**
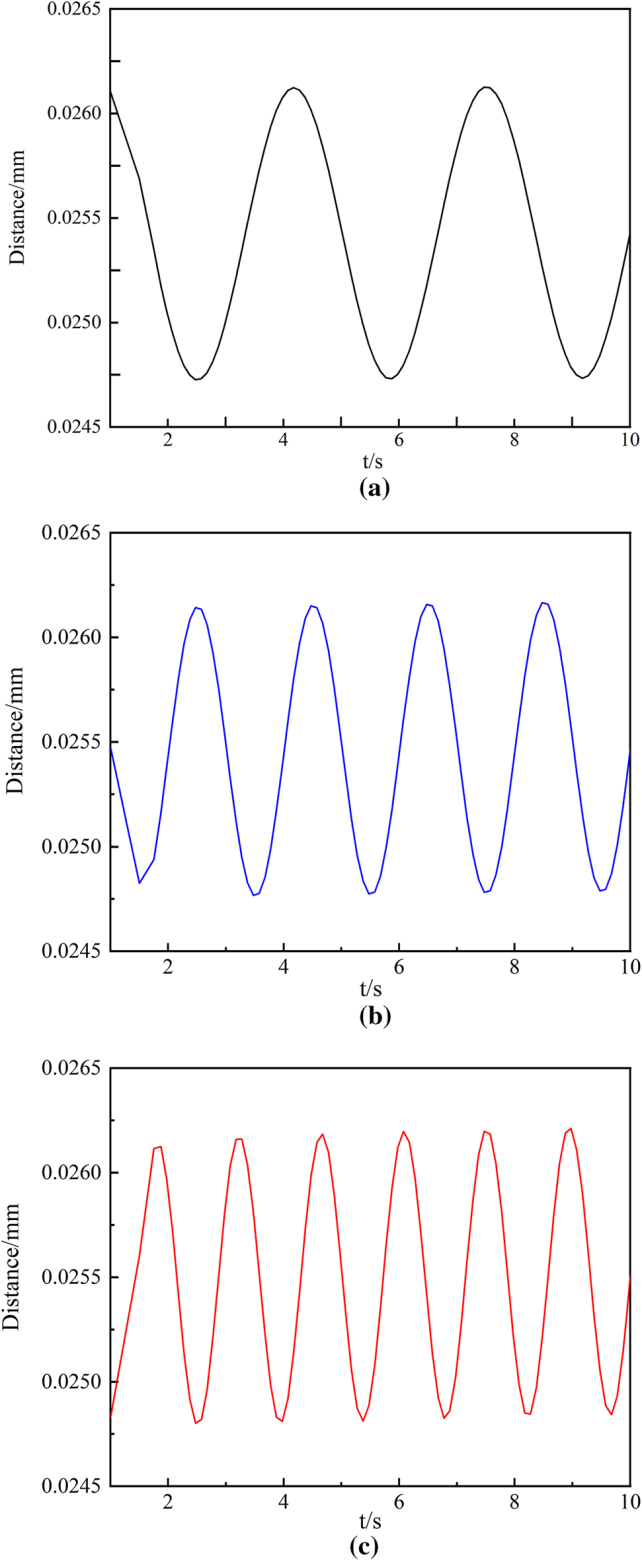
Vibration displacement of the stent at different respiratory frequencies **a** Normal respiratory rate **b** Rapid respiratory rate **c** Acute respiratory rate

To study the influence of pressure generated by different breathing states on the vibration characteristics, a stent with a diameter of 14 mm was selected for vibration analysis. A periodic pressure with an amplitude of 0.007 MPa was applied to the outer wall of the trachea, and the frequencies were set to 0.3 Hz, 0.4 Hz, and 0.5 Hz for simulation analysis. The radial displacement curve of the stent was obtained, and the result is shown in Fig. 7. It could be seen from Fig. 7 that different breathing frequencies would lead to various displacement variation periods of the stent. The more frequently the suction, the faster the deformation of the stent. With the increase in respiratory rate, the radial displacement of the trachea also increased, but there was no significant difference in the maximum displacement. Obviously, when patients had asthma and other rapid breathing or intense exercise, significant transpulmonary pressure would occur in the lung, and the stent was more likely to be lost. Therefore, patients with airway stenosis should ensure smooth and normal breathing after stent placement. In addition, patients should provide a reasonable respiratory rate during exercise to avoid stent displacement.

In conclusion, the diameter, respiratory rate, and pressure of the trachea greatly influence the vibration characteristics of the stent. The larger the diameter of the diseased trachea, the more likely the stent will deform when external forces compress it. When the stent deforms to a certain extent, it will break away from the original support position, thus causing secondary damage to the tracheal wall. At the same time, in the process of treatment, patients should try to keep their breathing in a normal breathing state. Once the respiratory discomfort caused by external factors is frequent, patients should be examined in time. And the stent removal or reinsertion should be carried out when necessary to reduce the risk of stent loss to the patient’s safety.

### Modal analysis of tracheal stent

**Fig. 8:**
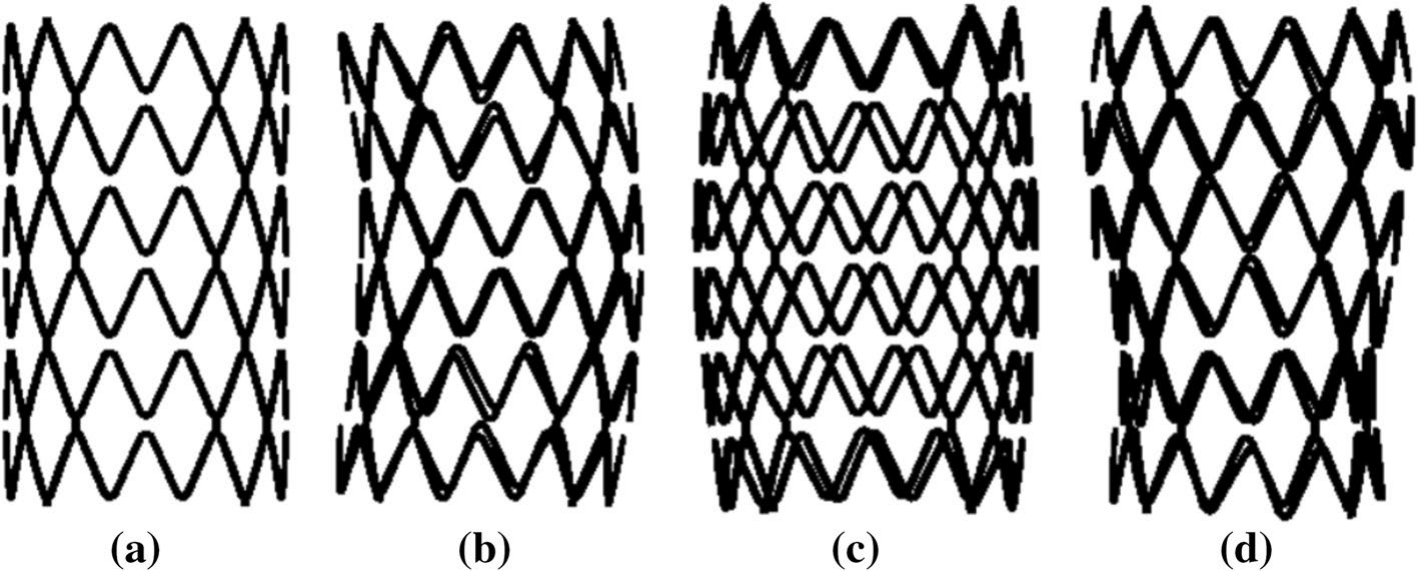
Vibration mode figure of tracheal stents **a** The first-order modal **b**. The third-order modal **c** The sixth-order modal **d**. The tenth-order modal

**Fig. 9:**
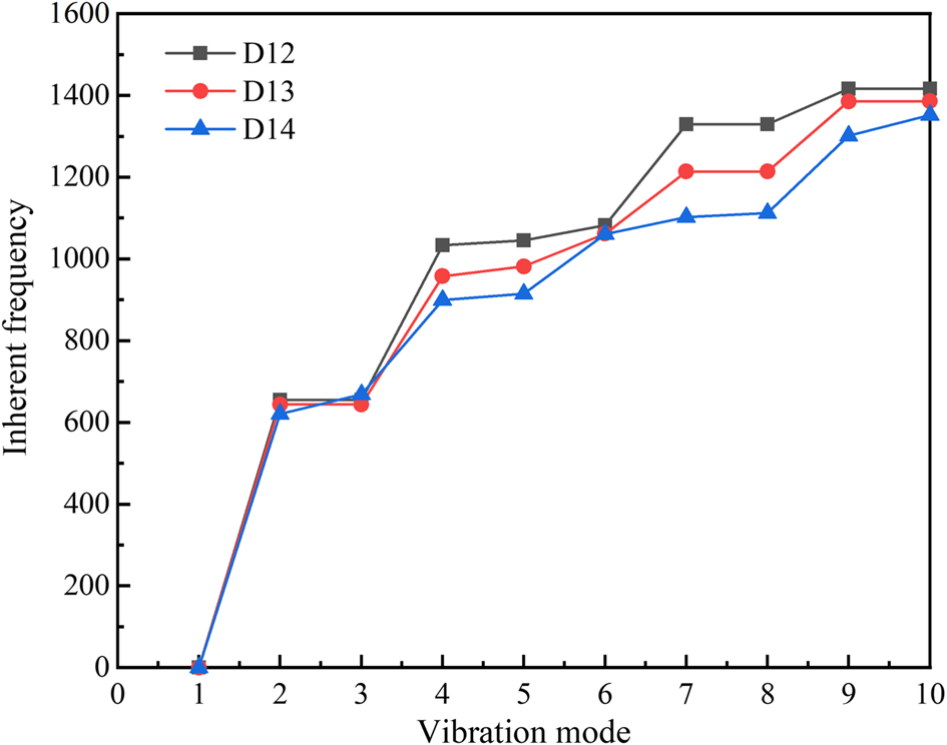
Natural frequency trend of different diameter tracheal stents

Modes are the inherent vibration properties of a structure, and each mode has a specific natural frequency, damping ratio, and mode shape. These modal parameters can be obtained by analysis software or test calculation [23]. Modal analysis is a modern method to study the dynamic characteristics of structures and the application of the system discrimination method in engineering vibration. Natural frequency is a physical property of an object, determined by its structure, size, shape, and other factors. This physical characteristic does not change whether the object is in a vibrational state. The ultimate goal of modal analysis is to identify the modal parameters of the system to provide a basis for the analysis of the vibration characteristics of the structural system, diagnosing and detecting vibration faults, and optimizing the structure. The vibration modes of the stent under different modes were obtained, as shown in Fig. 8. Besides, the natural frequencies of tracheal stents with different diameters were extracted, and the results are shown in Fig. 9.

It can be seen in Fig. 8 that with the increase of the mode, the radial deformation of the stent became larger. Since the cough frequency was small, the trachea and the stent would not resonate. Figure 9 shows that the larger the diameter of the stent, the smaller its natural frequency, which indicated that the stent with a larger diameter was prone to deformation when stimulated, thus resulting in a loss. Therefore, the supporting performance of the larger diameter stent should be optimized to enhance its anti-vibration performance.

### Random vibration analysis of tracheal stent

The tracheal stent is fixed to the inner trachea wall by friction and tension. The cross-sectional area of the trachea changes dramatically when spontaneous and induced cough occurs in humans. The smooth muscle of the trachea exerts a considerable radial pressure on the stent, resulting in nonlinear deformation of the stent [24, 25]. Due to the randomness of cough intensity, the stress and contractile force generated by cough fluctuate. Therefore, cough can be considered as random radial excitation, which increases the complexity of the dynamic study of the tracheal stent. Thus, the nonlinear dynamic equation of the Nitinol stent will be solved in this section, and the excitation parameters' influence on the stent's vibration performance is also analyzed.

Because it is difficult to simulate the random vibration of the tracheal stent system, this paper only uses Matlab software to numerically simulate the established nonlinear dynamic model and analyzes the influence of each parameter on vibration. During a cough, the amplitude of a cough cycle can be seen as a decaying process. That is, the amplitude of the cough is more prominent at the beginning. At this time, the radial deformation of the trachea is about 1–3 mm. With the decrease of cough intensity, the amplitude of the cough decreases to 0 until the next cough occurs. To simplify the calculation, the first-order natural frequency of the stent was set to 100, and the cough frequency was set to 1–5 Hz. And the frequency of sound associated with coughing was usually between 50 and 300 Hz [26] Since the average pressure of an adult male cough was between 15 and 157cm H_2_O [27], the stent length was 24 mm. The diameter of the trachea was 10–15 mm, and it could be calculated that the range of excitation force acting on the stent surface during cough was about 1–40 N. Due to the simplification of the nonlinear dynamic equation of the Nitinol stent model, the selection of the system damping is uncertain, and the parameters solved are not of great practical significance. Therefore, the Matlab numerical analysis method is used to explore the influence of various parameters on the stability of the stent system.

#### Influence of Duffing coefficient b value on vibration result

**Fig. 10:**
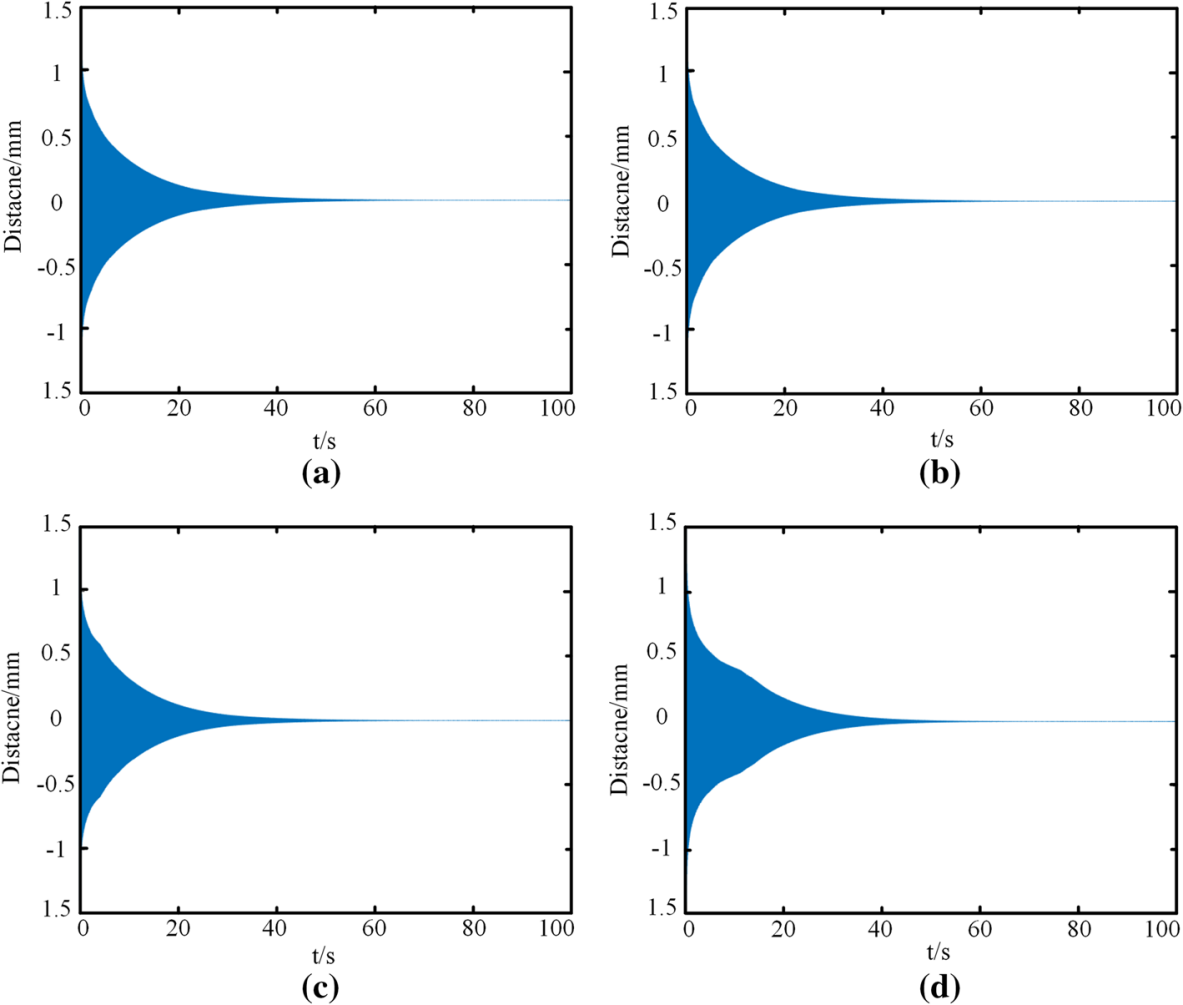
Time history diagram of tracheal stent system at different *b* values. **a** *b* = 1. **b** *b* = 10. **c** *b* = 100. **d** *b* = 1000

Duffing coefficient is the vibrator describing the forced vibration, and the larger its value is, the more frequent the vibration is. To explore the influence of coefficient on vibration stability in Eq. (22), a set of default parameters was provided, that was $\omega = 100$,e = 10,$\Omega = 5$, set the damping coefficient as $\eta_{1} = 0.1,\eta_{2} = - 1,\eta_{3} = 10$, and *b* values in the equation were adjusted to 1, 10, 100, and 1000 in Matlab software. The time history diagram of the tracheal stent is shown in Fig. 10.

As shown in Fig. 10, when subjected to external random excitation, the tracheal stent system always presented a trend of attenuation vibration and finally tended to a stable value. In addition, the maximum vibration displacement of the system is the same under different Duffing coefficients. When *b* value was less than 100, the stents tended to be stable for a long time, and the decay trend was steeper. However, when b value was 1000, the vibration attenuation trend of the tracheal stent began to become gentle within the 40 s, but the overall difference was not significant. Therefore, according to the numerical calculation results, for the original differential equation, when the value of b was changed, the tracheal stent system always performed attenuated vibration, which had little influence on the system's stability.

#### Influence of excitation amplitude *e* value on vibration result

**Fig. 11:**
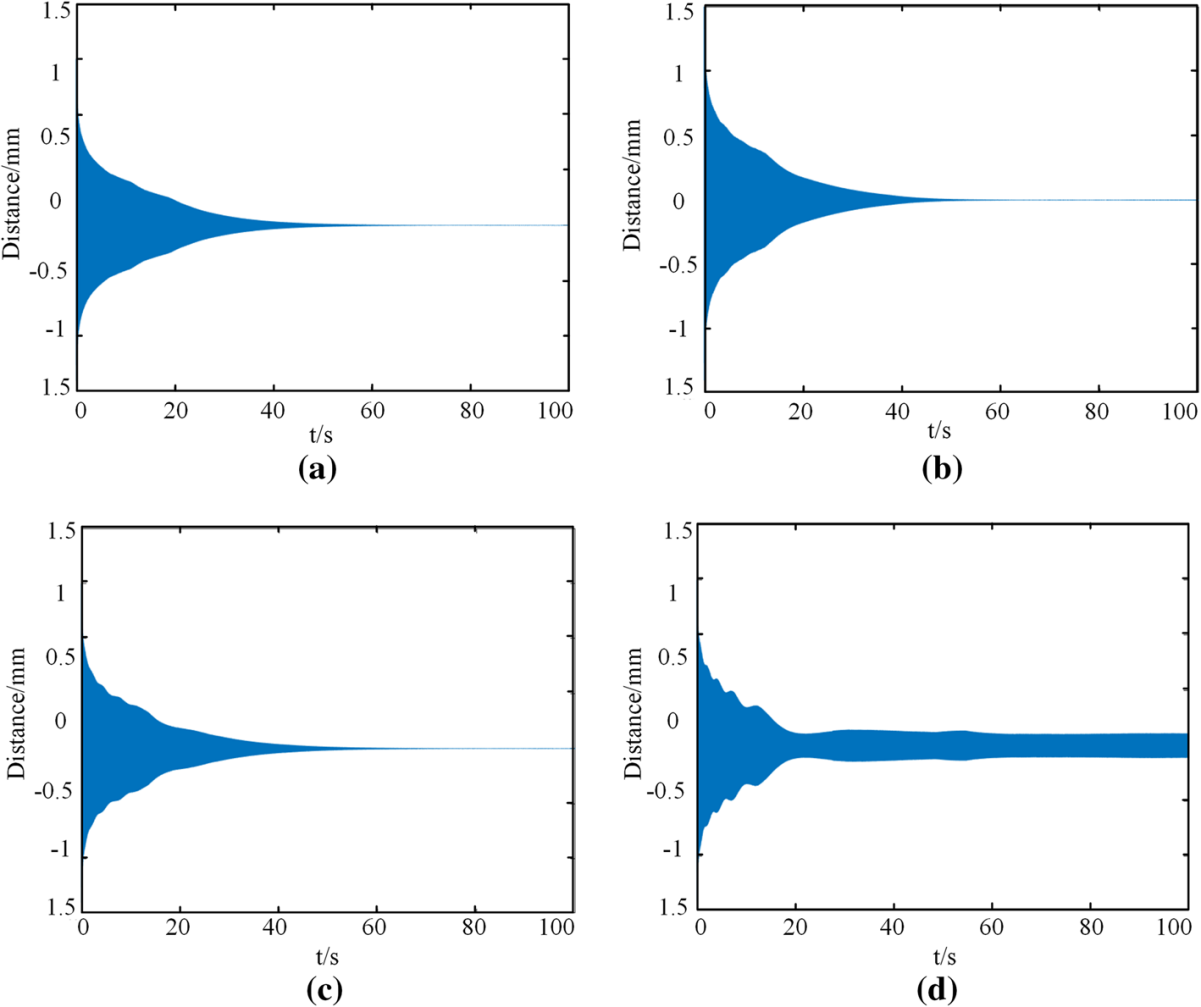
Time history diagram of tracheal stent under different excitation amplitudes. **a** *e* = 1. **b** *e* = 10. **c** *e* = 20. **d** *e* = 40

Cough intensity has great randomness, so the contraction force generated by the trachea during cough is also dynamic. To explore the influence of vibration caused by the excitation force on the stability of the tracheal stent system, *b* value was selected as 1000, and other parameters were fixed as the default values. The e values in the equation were adjusted to 1, 10, 20, and 40 N in Matlab software, and the time history diagram of the stent was obtained, as shown in Fig. 11.

As can be seen from Fig. 11, with the increase of the amplitude of the excitation force, the tracheal stent system gradually changed from an attenuated vibration to a stable vibration. When *e* value was small, the trend and time of vibration attenuation of the support were almost the same, and the radial displacement generated by the system almost approached 0 when it was stable. When the amplitude was large, the stent system quickly decayed to stable vibration, and the radial displacement of the tracheal stent in stable vibration was about 0.3 mm. This indicated that when the excitation force generated by the cough was too large, the tracheal stent system was still in a vibration state at the end of the first cough and entered into attenuation vibration again at the next cough. Therefore, when the excitation force was large, the more frequent the vibration of the stent was, the more likely it was to fall off.

#### Influence of frequency value of excitation force Ω on vibration result

**Fig. 12:**
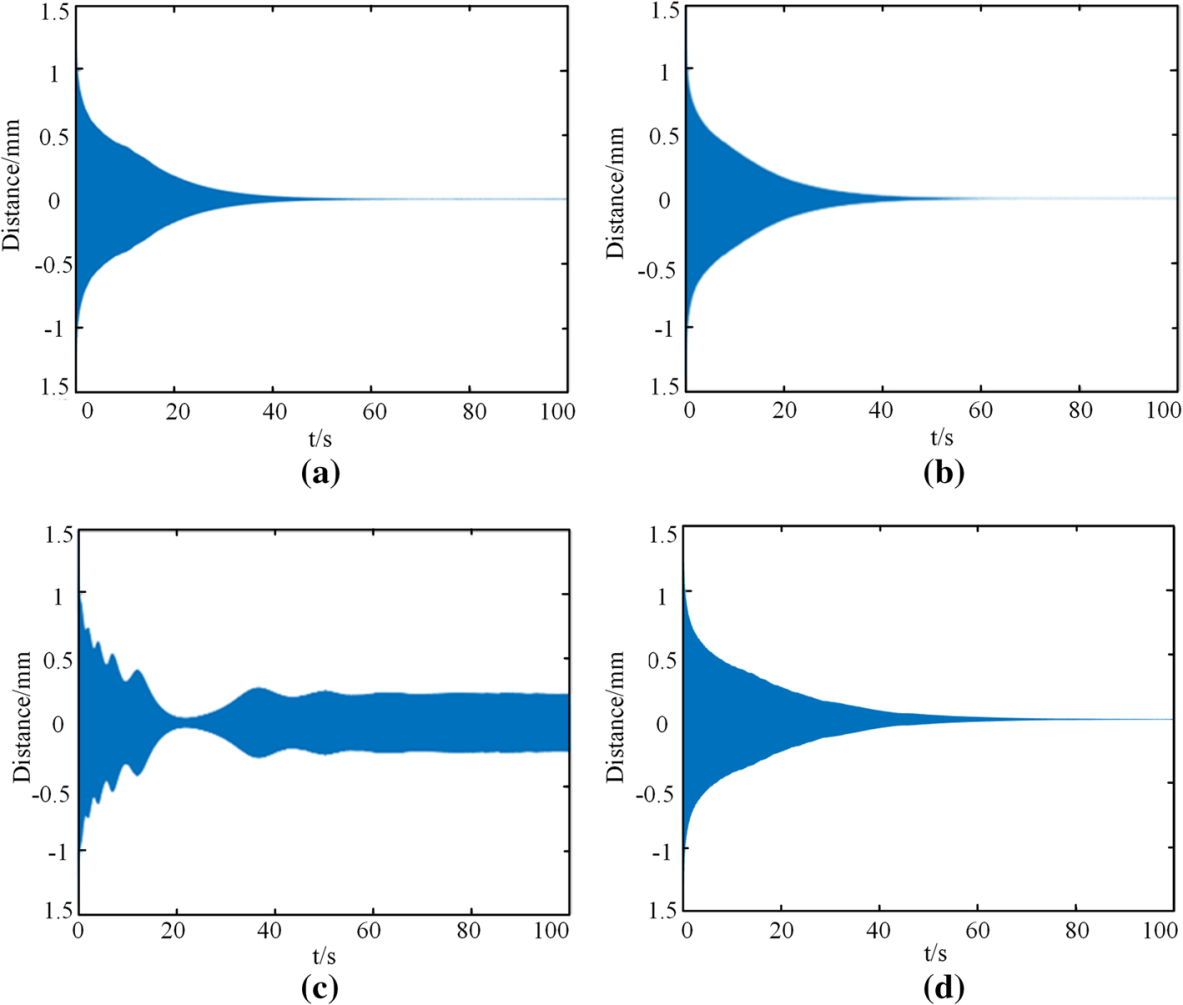
Time history diagram of the tracheal stent under different excitation frequencies. **a** Ω = 5. **b** Ω = 100. **c** Ω = 200. **d** Ω = 300

A cough will cause the airway to vibrate at a certain frequency, and the frequency of the cough varies among people of different ages. Children's breathing efficiency is poor, alveolar gas exchange is insufficient, and their cough frequency is faster than adults. To better observe the influence of frequency on the stability of the tracheal stent, the amplitude was set as the maximum value, and the frequency of normal cough was 5 Hz for analysis. In addition, to discuss whether the system would produce resonance, the natural frequencies are numerically solved at one, two, and three times (i.e., 100 Hz, 200 Hz, 300 Hz). The time history of the tracheal stent under these conditions is shown in Fig. 12.

As shown in Fig. 12, when the human body coughs normally, the scaffold system eventually becomes stable from the vibration state. Besides, for the tracheal stent system, the magnitude of excitation frequency had no significant influence on the stent stability. The solution of the original differential equation was greatly influenced only when the excitation frequency was twice the natural frequency of the stent. At this time, the stent's vibration changed from attenuation to stable. This indicated that the resonance phenomenon would occur when the excitation frequency was about two times the natural frequency, but it would not happen under normal breathing conditions.

#### Influence of nonlinear damping coefficient on vibration result

**Fig. 13:**
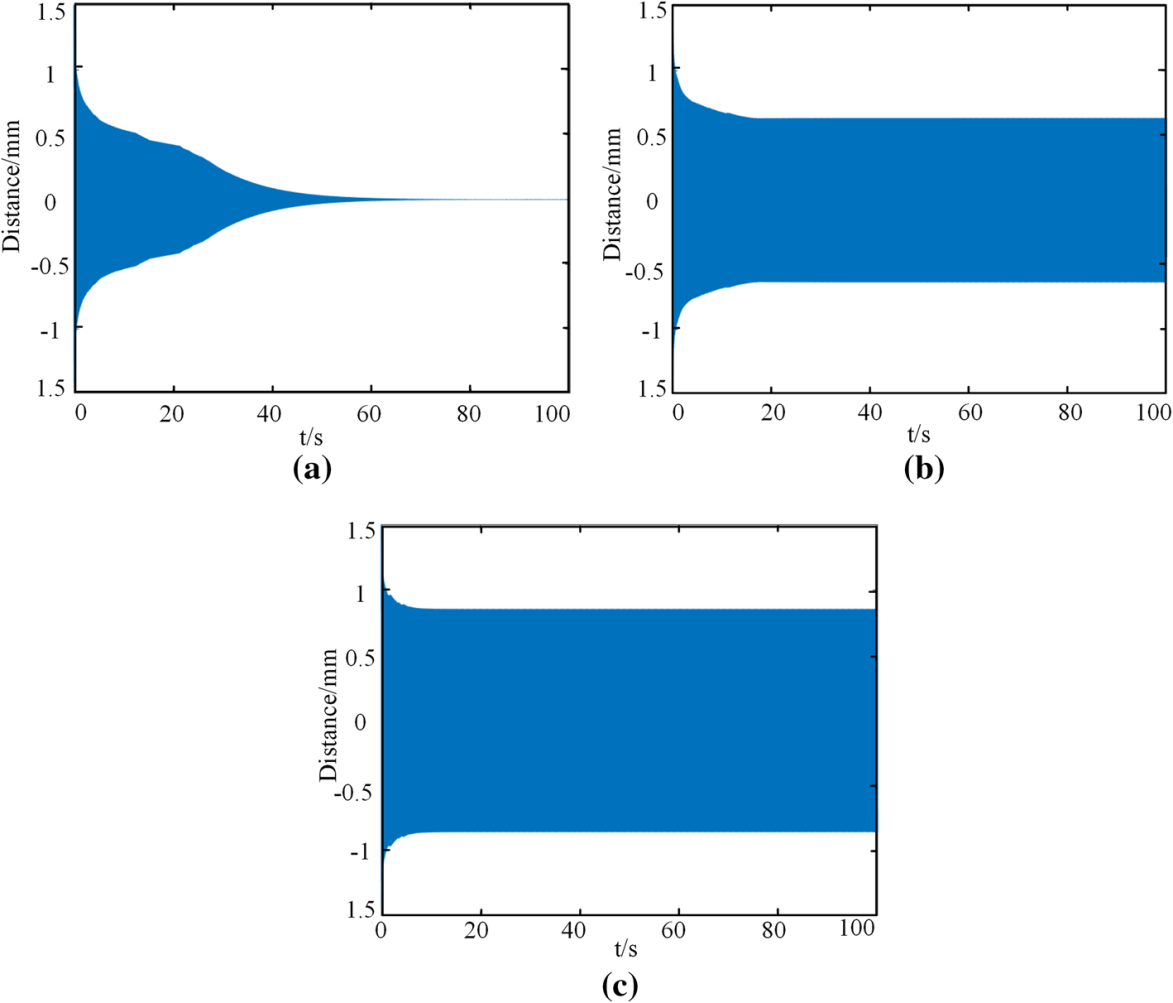
Time history diagram of the tracheal stent under different excitation amplitudes. **a** *η*_2_ = − 0.1. **b** *η*_2_ = − 1. **c** *η*_2_ = -10

From the established nonlinear dynamic equation, the nonlinear damping coefficient is mainly related to the parameters of the stent itself. The established tracheal stent system will significantly alter when a damping coefficient changes. To simplify the analysis, this paper only discussed the influence of the negative damping coefficient on vibration results. The excitation frequency was determined to be 5 Hz, the amplitude of the excitation force was determined to be 10 N, and other values were the default values. The negative damping coefficients were − 2, − 3, and − 4, and the time history of the stent is shown in Fig. 13.

As shown in Fig. 13, when the absolute value of the damping coefficient was small, the tracheal stent presented an attenuated vibration trend when excited. However, when the damping coefficient was more significant than 2, the stent would quickly decay to a stable value for stable vibration, and the resulting radial deformation did not change with time. Therefore, when the absolute value of the nonlinear damping coefficient was smaller, the tracheal stent system was more easily stable under random radial excitation, such as cough.

## Discussion

As a treatment for the tracheal disease, tracheal stents can quickly relieve dyspnea in patients with narrow airways. However, when the stent is implanted into the human body, it will not only be affected by the complex physiological environment in the body, resulting in deformation. It also is affected by random incentives, such as cough and sneezing, resulting in stent loss, which threatens patients’ safety. In this paper, the theoretical analysis and numerical simulation are combined to study the vibration characteristics of the stent under different conditions during service. This can help observe the deformation of the stent. According to the pressure on the tracheal stent, the vibration is divided into periodic and random. On one hand, the influence of tracheal diameter and respiratory parameters on the vibration performance of the stent was obtained by stress–strain analysis of the tracheal stent coupling system. On the other hand, Matlab software was used to analyze the stability of the tracheal stent system and explore the possibility of stent resonance. What is more, the results can help doctors predict the likelihood of stent displacement and provide theoretical guidance for patients' postoperative care.

(1) The breathing process of the human body can be regarded as a periodic movement. The transpulmonary pressure created by the lungs during breathing directly acts on the tracheal wall, causing tracheal constriction. To explore the effect of tracheal contraction on stent movement in normal, rapid, and severe breathing, a periodic pressure was applied to the outer tracheal wall in the simulation, which was helpful in observing the radial deformation of the stent under different trachea diameters, respiratory pressures, and frequencies. The results showed that when the tracheal stenosis occurred in the larger grade of the trachea, the tracheal stent was more likely to fall off during the treatment. With the increase in respiratory rate and pressure, the radial displacement caused by the stent compression was also more prominent. This indicated that when the patients were breathing rapidly or exercising vigorously, the tracheal wall was often under high pressure, which increased the stent deformation. Therefore, patients should try to ensure stable breathing and avoid strenuous exercise during stent implantation. At the same time, when patients suffer from shortness of breath caused by other diseases or external factors and thus have discomfort, a medical examination should be conducted in time, and the stent should be removed when necessary. In addition, the stress cloud diagram of the trachea tissue shows that most of the contact pressure between the stent and the trachea wall during service is concentrated at the front and rear of the stent. When the stress is too large, the two ends of the stent are prone to granulation and other phenomena. Therefore, stable breathing also reduces the difficulty of stent removal.

(2) To understand the influence of the stent structure on the vibration performance, the modes of the stent with different diameters were firstly solved. The results showed that the larger the diameter, the smaller the natural frequency of the stent under the same vibration mode. These results indicate that the performance of the stents can be improved by improving the structure and optimizing the structural parameters, thereby reducing the influence of vibration on stent stability. In addition, due to the randomness of cough intensity, the pressure and contraction force fluctuate so that the cough can be considered random radial excitation. This paper used Matlab software to solve the established system differential equation and by adjusting the parameters to explore its influence on the system stability. Through analysis, it was found that when the tracheal stent was subjected to random excitation, it would produce a large radial displacement and attenuation vibration until the next cough. In addition, it was found that the Duffing coefficient b value and the frequency of normal cough could hardly affect the system stability. At the same time, the excitation force and damping coefficient had a significant influence on the system stability. When the excitation force amplitude of the cough exceeded the critical value of 20, the stent system changed from attenuated to stable periodic vibration. When the pressure generated by the cough was too enormous, the stent was prone to fall off.

The next step of this study is to simulate the random vibration characteristics of the tracheal stent based on a numerical solution and give a reasonable range of stent migration. At the same time, the relationship between stent displacement and the human cough mechanism will be established to help better doctors predict the possibility of stent loss. On the other hand, the structural characteristics of the tracheal stent will be comprehensively analyzed to explore the influence of different parameters on the vibration performance to provide a theoretical basis for the clinical use of stents and provide a direction for the optimal design of stents.

## Conclusion

This study analyzed the forced vibration performance of the Nitinol tracheal stent. The radial deformation of the stent under different respiratory cycles was obtained, and the safety performance of the stent under random vibration such as cough was considered. The forced vibration characteristics of the stent under service conditions were comprehensively obtained, thus making up for the deficiency of the stent vibration theory. Besides, the forced vibration characteristics of the stent under service conditions were obtained comprehensively, which could make up for the lack of stent vibration. However, this study still has some limitations.

(1) The tracheal stent showed interference with the tracheal tissue after implantation, and there was a specific contact force between the stent and the tracheal tissue. To simplify the analysis, only the normal contact state between the trachea and the stent was considered in the simulation, and the influence of contact force was ignored. (2) Tracheal tissue has complex material properties, and only the hyperelasticity of tracheal tissue was considered in this study. However, after stent placement, the viscoelasticity and anisotropy of the trachea would also affect the vibration characteristics of the stent. Therefore, it is necessary to establish the material properties of the intact trachea and consider the vibration properties of the stent at the cartilage in the future.

This study provides relatively more comprehensive and reliable puncture mechanics data over the experiment, which contributes to further research of tracheal biomechanical characteristics at all levels, guiding the design and manufacture of artificial tracheal grafts and the precise reconstruction of diseased airway sites, offering necessary data reference for clinical analysis of tracheal diseases, and providing the theoretical basis for the virtual interventional surgery development.

## Methods

As a usual medical device, the tracheal stent can be implanted in the position of tracheal stenosis to support the trachea wall, ensuring the patient's smooth breathing [28]. From the working principle of the tracheal stent, it is clear that the stent is an interference fit with the tracheal wall when it provides lasting support for the diseased trachea. And the stent will be constrained by the gas tube wall during service. Therefore, the tracheal wall movement will significantly impact the coupling system of the trachea and stent, resulting in forced vibration of the trachea stent. Considering that the tracheal stent will be subjected to various forms of impact when working in the human body, this paper analyzes the forced vibration of the stent into two aspects: periodic vibration and random vibration.

### Establishment of the airway and tracheal stent models

**Fig. 14:**
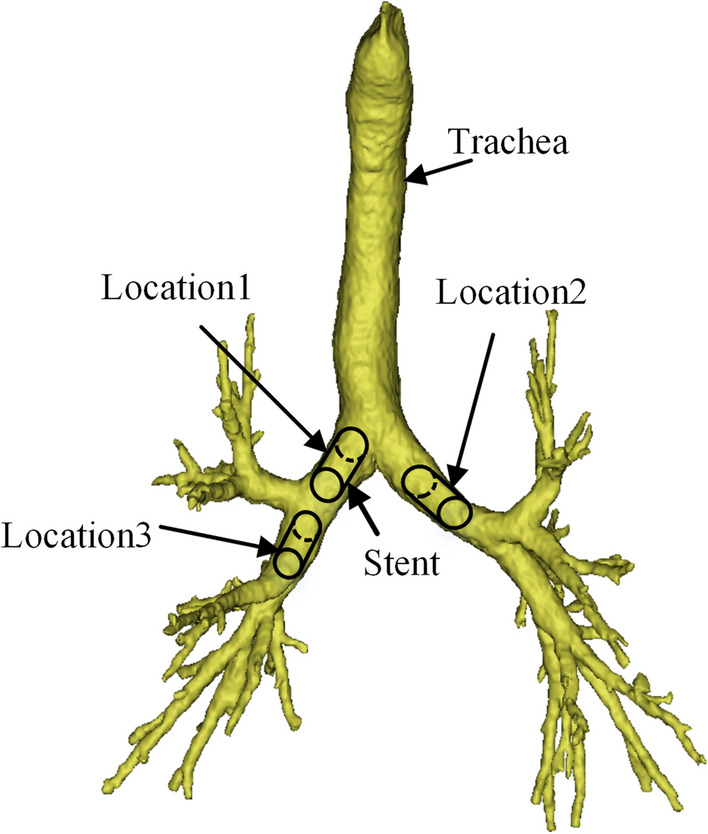
Reconstructed models of airways

To build a 3D model of the stent, the diameter and length of the trachea need to be determined. In this section, the trachea model was established by a three-dimensional reconstruction method, and the trachea model was measured to obtain the required data. A 256-row spiral scan was performed from the top of the central trachea to the level of the proline muscle in an adult male patient with a thickness of 0.5 mm. The CT scan data were saved in DICOM format and imported into Minics21 software. The final step was to complete the three-dimensional reconstruction of the trachea and bronchial tree model. According to research, airway stenosis occurs mainly in the main bronchus and lower trachea. To simplify the analysis, three positions of left main bronchus, right main bronchus, and right secondary bronchus were selected for simulation analysis, as shown in Fig. 14. Mimics21 software measured the diameter, cross-sectional area, and length of the above three positions. The primary parameter data are shown in Table 2.

**Table 2:** Statistical table of measurement data at different positions of the trachea

Location	Diameter (mm)	Cross-sectional area (mm)	Length (mm)
1	14.06	0.04	22.25
2	13.06	0.03	4.20
3	12.08	0.11	33.22

**Fig. 15:**
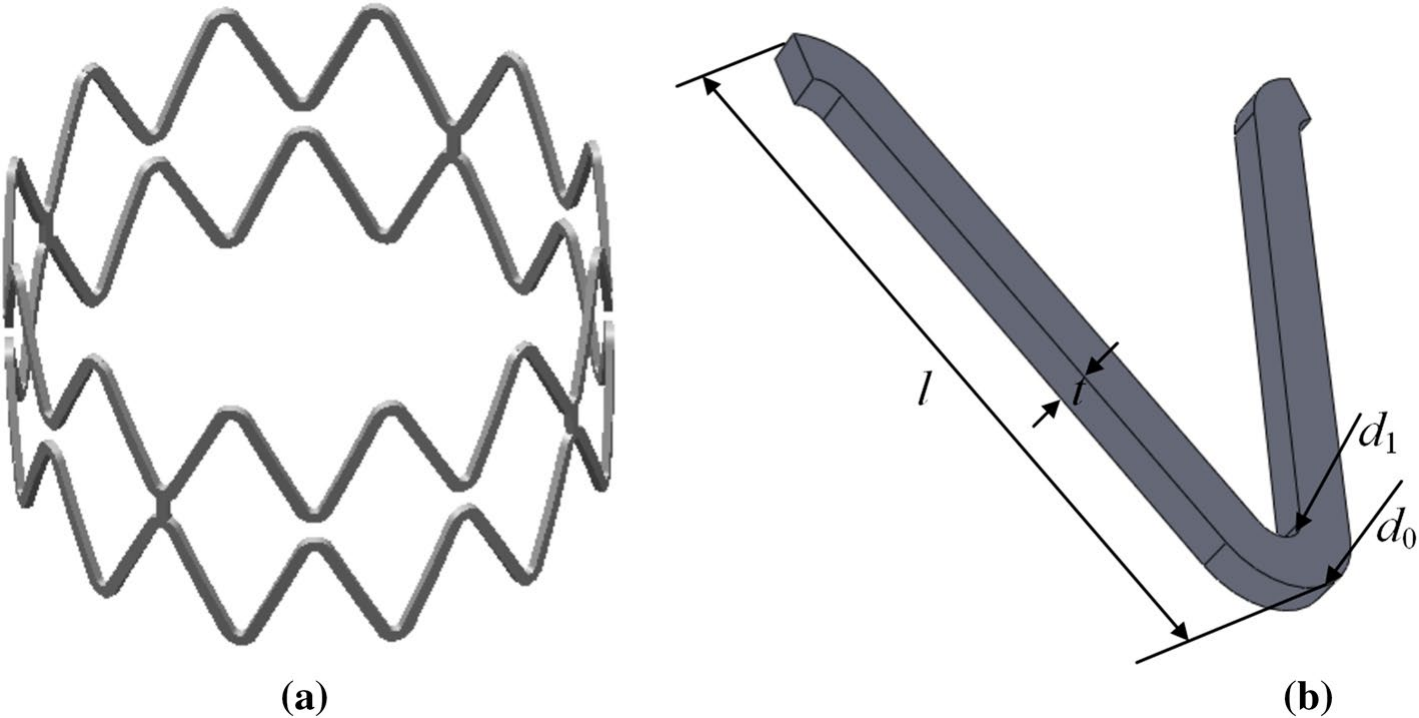
Structure diagram of the tracheal stent unit. **a** Three-dimensional model of the stent. **b** Structure diagram of V-shaped element

Medical Nitinol stents were selected for vibration analysis in this paper. According to the measured tracheal data in Table 1, the diameter of the medical Nitinol V-shaped stent was determined to be 12, 13, and 14 mm, and the corresponding model was established in Solidworks software. Taking the stent with a diameter of 14 mm as an example, the unit structure is shown in Fig. 15, and the detailed size of the stent is shown in Table 3.

**Table 3:** Parameters of V-shaped Nitinol tracheal stent

Parameter	Data (mm)
Outer arc diameter *d*_0_	0.45
Diameter of inner arc *d*_1_	0.3
Axial height of the stent body *l*	3.4
External diameter of the stent *D*	14
The total length of the stent *L*	24
The thickness of the stent *t*	0.2
Number of circumferential units	12
Number of axial units	6

### Analysis of nitinol material properties and trachea biomechanical properties

Nitinol has good shape memory and biocompatibility. It can withstand very large deformation during loading and recover quickly after unloading. Nitinol material shows austenitic phase (A) without loading. When the load exceeds particular stress, the austenitic phase (A) transforms into the martensite phase (M), generating a large amount of strain. After unloading, Nitinol changes from the martensite phase (M) to the austenite phase (A) [29]. The variation of the strain of Nitinol material with temperature is nonlinear. According to the functional relationship between the force and strain at a fixed temperature, the stress and strain function of memory alloy can be deduced as follows:

1 $$ \sigma = x_{1} \varepsilon + x_{2} \varepsilon^{2} + x_{3} \varepsilon^{3} + \left( {x_{4} \varepsilon + x_{5} \varepsilon^{2} + x_{6} \varepsilon^{3} + x_{7} \varepsilon^{4} } \right)\dot{\varepsilon }, $$

where $x_{i}$ is the coefficient; $\sigma$ is the stress; $\varepsilon$ is the strain.

The material properties of tracheal tissue are complex, including hyperelasticity, anisotropy, and viscoelasticity [30]. At present, constitutive models to describe the deformation of elastic bodies have been widely used in soft tissue research [31, 32]. In this section, the hyperelastic Mooney-Rivlin model is used to describe the biomechanical properties of the trachea.

The stress–strain relationship of trachea tissue materials is usually nonlinear, it can be regarded as incompressible, isotropic, and hyperelastic materials. For this nonlinear elastic material, the constitutive relation can be derived from the scalar strain energy function *W*, the strain energy function *W* is a function of deformation.

2 $$ W = W(I_{1} ,I_{2} ,I_{3} ), $$

where $I_{1}$, $I_{2}$, and $I_{3}$ are, respectively, the first, second, and third basic invariants of soft tissue deformation tensor.

Since the trachea material is treated as isotropic, the strain or deformation measurement needs to be independent of the selected coordinate system, the right Cauchy-Green deformation tensor *C* provides such a strain measure, because it is derived from the deformation gradient *F*, that is *C* = *F*^*T*^*F* [33]. The invariant of Cauchy stress tensor means that there exists an inclined plane in the coordinate space with only normal stress but no shear stress, it can be expressed as

3 $$ \left( {\sigma_{ij} - \sigma \delta_{ij} } \right)n_{j} = 0, $$

where $\sigma_{ij}$ is stress components in all directions, $\sigma_{ij}$ is strain components in all directions, and $n_{j}$ is principal stress direction.

The above formula is expanded to get

4 $$ \left\{ {\begin{array}{*{20}r} \hfill {\left( {\sigma_{x} - \sigma } \right)l + \tau_{xy} m + \tau_{xz} n = 0} \\ \hfill {\tau_{yx} l + \left( {\sigma_{y} - \sigma } \right)m + \tau_{yz} n = 0} \\ \hfill {\tau_{zx} l + \tau_{zy} m + \left( {\sigma_{z} - \sigma } \right)n = 0} \\ \end{array} } \right.. $$

The main plane is a plane with only normal stress and no shear stress, and the normal stress on the main plane is called the main stress, and the normal direction of the main plane is the main direction. Equation 4 is a linear homogeneous equation with respect to the principal direction (*l*, *m*, *n*). The condition that it has a nonzero solution is that the determinant of the coefficient is 0:

5 $$ C = \left| {\begin{array}{*{20}c} {\sigma_{x} - \sigma } & {\tau_{xy} } & {\tau_{xz} } \\ {\tau_{yx} } & {\sigma_{y} - \sigma } & {\tau_{yz} } \\ {\tau_{zx} } & {\tau_{zy} } & {\sigma_{z} - \sigma } \\ \end{array} } \right| = 0. $$

It can be obtained by solving the characteristic equation of the stress tensor:

6 $$ \left\{ \begin{gathered} I_{1} = {\text{tr}} ({\text{C}}) = \lambda_{1}^{2} + \lambda_{2}^{2} + \lambda_{3}^{2} \hfill \\ I_{2} = \frac{1}{2}\left( {({\text{tr}} {\text{C}})^{2} - {\text{tr}} \left( {{\text{C}}^{2} } \right)} \right) = \lambda_{1}^{2} \lambda_{2}^{2} + \lambda_{2}^{2} \lambda_{3}^{2} + \lambda_{3}^{2} \lambda_{1}^{2} \hfill \\ I_{3} = \det ({\text{C}}) = \lambda_{1}^{2} \lambda_{2}^{2} \lambda_{3}^{2} = J^{2} \hfill \\ \end{gathered} \right., $$

where $\lambda_{1}$, $\lambda_{2}$, and $\lambda_{3}$ are the eigenvalues of *C*.

For a given state of stress, the size and direction of principal stress is determined, it does not change along with the transformation of coordinate system. The strain energy function can be divided into deviation part and expansion part, as shown by the following formula:

7 $$ W = W_{{\text{deviatoric }}} \left( {I_{1} ,I_{2} } \right) + W_{{\text{dilatational }}} (J), $$

where $I_{1}$, $I_{2}$ are determined by the formula $I_{p} = J^{ - 2/3} I_{p}$; J is the ratio of the volume of material to the volume of undeformed material; $W_{{\text{deviatoric }}}$ is the strain energy function of shape change; $W_{{\text{dilatational }}}$ is the strain energy function of volume change.

The Mooney-Rivlin model can simulate the deformation behavior of the hyperelastic materials by expressing the equations as power series. The following equation gives the two-parameter Mooney-Rivlin model:

8 $$ W = C_{10} \left( {I_{1} - 3} \right) + C_{01} \left( {I_{2} - 3} \right) + \frac{1}{d}\left( {J_{el} - 1} \right)^{2} $$

where $C_{01}$ and $C_{10}$ are the material parameters of the Mooney-Rivlin constitutive model.

Establishment of coupling vibration model of the trachea and stent under periodic vibration.

**Fig. 16:**
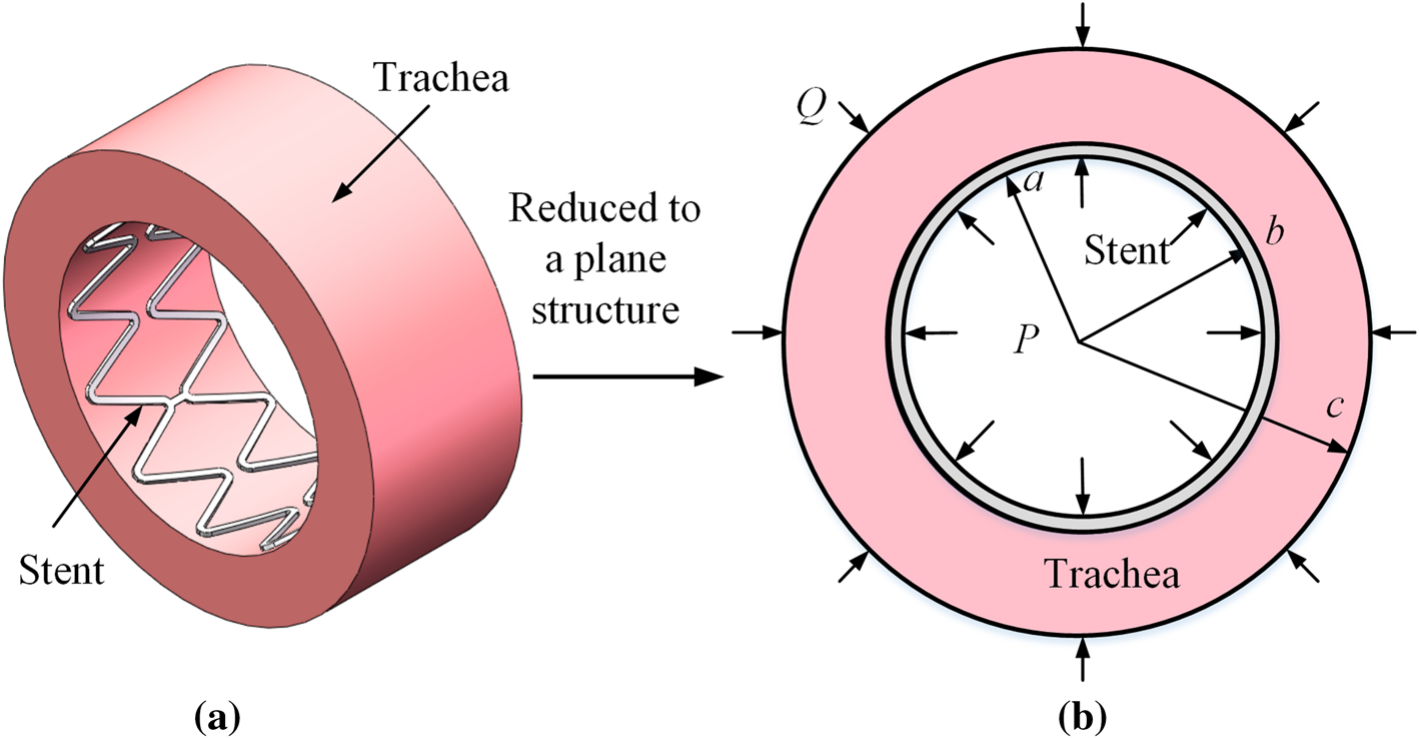
Coupling model of the trachea and stent

Human respiration is a passive process. Compression movement of the intercostal and diaphragmatic muscles causes gas to enter the lungs. Intrapulmonary and intrathoracic pressure will produce a pressure acting on the airway wall, that is, transpulmonary pressure, which will lead to the trachea movement and force the stent to vibrate. Human respiration can be seen as a sinusoidal periodic motion. In addition, breathing can be divided into normal, rapid, and violent breathing during strenuous exercise according to different states. Different breathing conditions also result in different pressures and frequencies applied to the trachea wall. Therefore, a simplified model of the trachea and stent system has been presented in this paper, as shown in Fig. 16. The trachea was regarded as a viscoelastic body, the natural frequency of the stent vibration was solved, and the characteristics of its forced vibration were analyzed.

The stent deformation can be approximated as a plane strain problem. That is, $\varepsilon_{z} = 0$. This section assumes as follows: (1) axisymmetric deformation: $u_{\theta }^{(2)} = 0,\tau_{r\theta }^{(2)} = 0$; (2) No strain in the radial direction of the stent: $\varepsilon_{r}^{(2)} = \partial u_{r}^{(2)} /\hat{O}r = 0$. Thus, $u_{r}^{(2)} = U(t),\varepsilon_{\theta }^{(2)} = U(t)/r$. The equilibrium equation can be obtained as follows:

9 $$ \rho (b - a)\ddot{U} + \frac{(a - b)(\alpha - \beta )}{{ab}}U + (q - p) = 0, $$

where $\alpha ,\beta$ is the proportionality constant, which is related to the damping ratio of the mode shape; $\rho$ is the density; *a* is the inner diameter of the tracheal stent; *b* is the outside diameter of the stent; *c* is the outer diameter of the trachea; and *p* is load.

For the trachea, it is regarded as an axially symmetric thick-walled tube subject to uniform internal pressure, considering the plane strain problem. The trachea is regarded as Kelvin viscoelastic material due to its viscoelasticity. According to the corresponding elastic–viscoelastic principle [34], the radial displacement of the tracheal plane strain is Laplace transformed, and inverse transformed, and the following results are obtained:

10 $$ u_{r} (r,t) = \frac{{q_{0} b^{2} }}{{c^{2} - b^{2} }}\left[ {\left( {\frac{3r}{{6k + G}}} \right)\left( {1 - e^{ - \beta t} } \right) + \left( {\frac{{c^{2} }}{Gr}} \right)\left( {1 - e^{{ - \frac{G}{\eta }t}} } \right)} \right], $$

where $G,\eta$ are elastic modulus and viscosity coefficient in the Kelvin shear model, respectively, $\beta = \left( {6k + G} \right)/\eta$; *k* is the bulk elastic modulus; *r* is the radius of the trachea; and *t* is time. According to the characteristics and boundary conditions of the Kelvin model, it can be obtained:

11 $$ q(t) = \left( {1 - b^{2} /c^{2} } \right)\left[ {U/\left( {\frac{3r}{{6k + G_{1} }} + \frac{{c^{2} }}{{G_{1} b}}} \right) + \frac{{\eta_{1} }}{{3\left( {1 + b^{2} /c^{2} } \right)b}}\dot{U}} \right]. $$

By substituting formula (11) into formula (9), the vibration differential equation of the coupled model can be obtained:

12 $$ M\ddot{u}_{r} + C\dot{u}_{r} + Ku_{r} = f(t), $$

where *M* is equivalent mass, $M = \rho_{2} (b - a)$; *C* is equivalent damping, $C = \frac{{\left( {c^{2} - b^{2} } \right)\eta_{1} }}{{3b^{3} + c^{2} b}}$; *K* is the equal coefficient of elasticity $K = \frac{(a - b)(\alpha - \beta )}{{ab}} + \frac{{c^{2} - b^{2} }}{{c^{2} \left( {\frac{3r}{{6k + G_{1} }} + \frac{{c^{2} }}{{G_{1} b}}} \right)}}$; and $f(t) = p(t)$.

In the forced vibration with damping of one degree of freedom, the natural frequency is $\omega = \sqrt {K/M}$. Since human breathing can be regarded as a sinusoidal law, the pressure of the tracheal wall to the stent is assumed to be $q(t) = p_{0} + \vartriangle p\sin \Omega t$ a pressure difference. Then the solution of Eq. 12 is

13 $$ u_{r} (t) = Ae^{ - \delta t} \sin \left( {\sqrt {\omega_{0}^{2} - \delta^{2} } t + \alpha } \right) + B\sin (\Omega t - \phi ) + \frac{{p_{0} }}{K}, $$

where *A, B* are amplitudes under different conditions; $\delta$ is damp; $\phi$ is phase angle; and $\Omega$ is the circular frequency of vibration.

According to the formula, the vibration is composed of two parts. The first part is the influence of damped free oscillation, whose amplitude will decay with time. The second part is the damped forced vibration, which is the vibration caused by the periodically changing exciting force. This paper considers the damped forced oscillation of the stent:

14 $$ u_{r} (t) = B\sin (\Omega t - \phi ) + \frac{{p_{0} }}{K}. $$

From formula (14), the radial displacement of the tracheal stent under vibration is related to the pressure on the stent, the frequency of the force, and the diameter of the stent.

Establishment of random vibration model of the stent under unexpected load excitation.

A cough is a sudden, often repetitive, spastic contraction of the chest cavity. Its primary physiological function is to remove mucus and foreign particles that enter the lungs during normal breathing. When the tracheal stent was implanted in the human body, it would produce muscular discomfort for a period, clinically manifested as recurrent cough and dyspnea. From a mechanical point of view, the trachea's cross-section changes dramatically during coughing. The trachea's smooth muscle contracted and relaxed violently, exerting enormous radial pressure on the trachea stent. In this case, the vibration response of the stent could be regarded as a random vibration process. The large vibration amplitude during cough will lead to the deformation or displacement of the tracheal stent, which will affect the treatment effect and even the risk of stent loss. Therefore, studying the nonlinear dynamic characteristics of Nitinol tracheal stent under radial excitation is necessary.

**Fig. 17:**
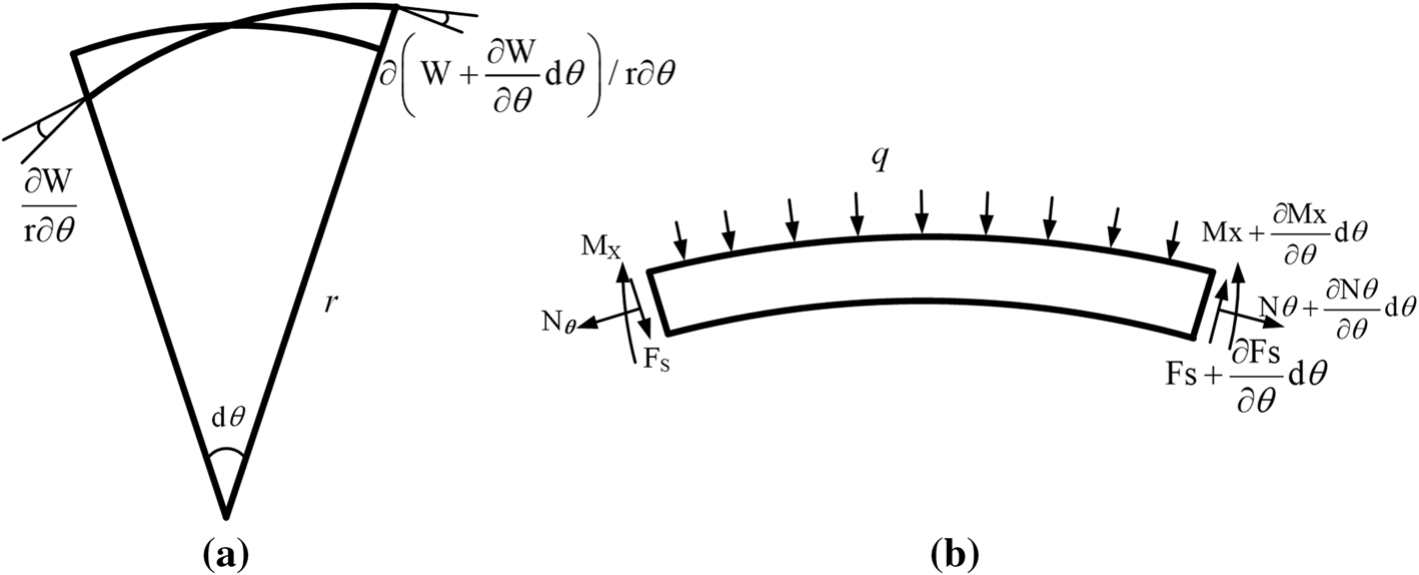
Tracheal stent force diagram

The Nitinol tracheal stent is assumed to be a thin-walled cylinder structure, and the plate and shell theory research method is used to carry out the force analysis [35]. The force of the tracheal stent is shown in Fig. 17.

According to Fig. 17 (a), the angle between the two ends is

15 $$ d\theta + \frac{\partial w}{{r\partial \theta }}\left( {w + \frac{\partial w}{{\partial \theta }}d\theta } \right) = \left( {1 - \frac{{\partial^{2} w}}{{r\partial \theta^{2} }}} \right)d\theta , $$

where $w$ is the stent deflection; *r* is the radius of the stent; and $\theta$ is the angle of the pillar unit.

It can be obtained by static analysis of Figure 17 (b):

16 $$ \left\{ {\begin{array}{*{20}l} {\frac{{\partial F_{s} }}{\partial \theta }d\theta + qrd\theta - N_{\theta } \left( {1 - \frac{{\partial^{2} w}}{{r\partial \theta^{2} }}} \right)d\theta = 0} \hfill \\ {\frac{{\partial M_{x} }}{\partial \theta }d\theta + F_{s} rd\theta = 0} \hfill \\ \end{array} } \right.. $$

Since the ratio of the thickness *h* to the radius *r* of the tracheal stent was far less than 1, that is *h/r* <  < 1, according to the allowable accuracy of the project––5/1000 < *h/r* < 1/5, it is assumed as follows:

(1) No rotation occurs in the XZ and XY planes; (2) The shear stress in the Z plane is assumed to be 0; (3) Ignoring the change of the normal displacement of plates and shells along the thickness.

Therefore, the dynamic model of Nitinol tracheal stent under radial load is

17 $$ \rho hr\frac{{\partial^{2} w}}{{\partial t^{2} }} = rq(\theta ,t) - \frac{{\partial^{2} M_{X} }}{{r\partial \theta^{2} }} - N_{\theta } \left( {1 - \frac{{\partial^{2} w}}{{r\partial \theta^{2} }}} \right). $$

The above dynamic equation can be obtained by applying plate and shell theory:

18 $$ \left\{ {\begin{array}{*{20}l} {M_{x} = \int_{{ - \frac{h}{2}}}^{\frac{h}{2}} y \cdot \sigma dy} \hfill \\ {N_{\theta } = \int_{{ - \frac{h}{2}}}^{\frac{h}{2}} \sigma dy} \hfill \\ \end{array} } \right., $$

where $y$ is the distance of collocation neutral layer; *σ* is the circumferential stress.

According to the geometric relation, the relation of the circumferential strain *ε* can be expressed as $\varepsilon = \frac{w}{r + y},y < < r$, which does not depend on $y$.

Therefore, in the above equation $M_{X} = 0,N_{\theta } = h\sigma$, substituting Eq. (1) into the Eq. (17), the dynamic response equation of Nitinol tracheal stent under radial random excitation can be obtained as follows:

19 $$ \rho hr\frac{{\partial^{2} w}}{{\partial t^{2} }} = rq(\theta ,t) - h\left[ {\frac{{x_{1} }}{r}w + \frac{{x_{2} }}{{r^{2} }}w^{2} + \frac{{x_{3} }}{{r^{3} }}w^{3} + \dot{w}\left( {\frac{{x_{4} }}{{r^{2} }}w + \frac{{x_{5} }}{{r^{3} }}w^{2} + \frac{{x_{6} }}{{r^{4} }}w^{3} + \frac{{x_{7} }}{{r^{5} }}w^{4} } \right)} \right]\left( {1 - \frac{{\partial^{2} w}}{{r\partial \theta^{2} }}} \right). $$

Obviously, the radial load was denoted by $w(\theta ,t)$ in this formula the periodic function was denoted by $q(\theta ,t)$, $\theta$ with the period $2\pi$. And the load of the tracheal wall on the stent is elastic and related to the deflection $w$ and time *t* of the stent. Based on the above conditions, the following assumptions were made:

20 $$ \left\{ {\begin{array}{*{20}l} {w(\theta ,t) = \sum\limits_{i = 1}^{\infty } {u_{i} } (t)\sin i\theta } \hfill \\ {q(\theta ,t) = w\tau (t) = \tau (t)\sum\limits_{i = 1}^{\infty } {u_{i} } (t)\sin i\theta } \hfill \\ \end{array} } \right. $$

If the:

21 $$ \begin{gathered} y = u_{k} ,e = \frac{1}{\rho h},\omega^{2} = \frac{{a_{1} }}{{\rho r^{2} }},b = \frac{{3\left( {a_{3} + k^{2} a_{2} } \right)}}{{4\rho r^{2} }} \hfill \\ \eta_{1} = \frac{{k^{2} }}{{\rho r^{2} }},\eta_{2} = \frac{{3\left( {a_{5} + k^{2} a_{4} } \right)}}{{4\rho r^{4} }},\eta_{3} = \frac{{5\left( {a_{7} + k^{2} a_{6} } \right)}}{{8\rho r^{6} }} \hfill \\ \end{gathered} $$

Substituting Eq. (21) into Eq. (19), Fourier series can expand all periodic functions into trigonometric functions. To reduce the workload, let $\tau (t) = \cos \Omega t$. Therefore, Eq. (19) can be written as

22 $$ \ddot{X} + \omega^{2} X = - bX^{3} - \left( {\eta_{1} + \eta_{2} X^{2} + \eta_{3} X^{4} } \right)\dot{X} + eX\cos \Omega t, $$

where $\omega$ is inherent frequency; *b* is the coefficient of the Duffing equation; $\eta_{1}$, $\eta_{2}$, $\eta_{3}$ are the damping coefficients; *y* is radial displacement; $\Omega$ is cough frequency; and t is time.

It can be inferred from Eq. (22) that, in the process of random vibration, the stability of the tracheal stent coupling system depends on the vibration frequency, the amplitude of the excitation force, and the nonlinear damping coefficient.

## Data Availability

Not applicable.
